# Implementation and analysis of a parallel kalman filter algorithm for lidar localization based on CUDA technology

**DOI:** 10.3389/frobt.2024.1341689

**Published:** 2024-02-02

**Authors:** Lesia Mochurad

**Affiliations:** Department of Artificial Intelligence, Lviv Polytechnic National University, Lviv, Ukraine

**Keywords:** extended kalman filter, lidar, CUDA technology, real-time systems, acceleration

## Abstract

**Introduction:** Navigation satellite systems can fail to work or work incorrectly in a number of conditions: signal shadowing, electromagnetic interference, atmospheric conditions, and technical problems. All of these factors can significantly affect the localization accuracy of autonomous driving systems. This emphasizes the need for other localization technologies, such as Lidar.

**Methods:** The use of the Kalman filter in combination with Lidar can be very effective in various applications due to the synergy of their capabilities. The Kalman filter can improve the accuracy of lidar measurements by taking into account the noise and inaccuracies present in the measurements.

**Results:** In this paper, we propose a parallel Kalman algorithm in three-dimensional space to speed up the computational speed of Lidar localization. At the same time, the initial localization accuracy of the latter is preserved. A distinctive feature of the proposed approach is that the Kalman localization algorithm itself is parallelized, rather than the process of building a map for navigation. The proposed algorithm allows us to obtain the result 3.8 times faster without compromising the localization accuracy, which was 3% for both cases, making it effective for real-time decision-making.

**Discussion:** The reliability of this result is confirmed by a preliminary theoretical estimate of the acceleration rate based on Ambdahl’s law. Accelerating the Kalman filter with CUDA for Lidar localization can be of significant practical value, especially in real-time and in conditions where large amounts of data from Lidar sensors need to be processed.

## 1 Introduction

Lidar, or light and range detection, is a method of remote sensing (Tian et al., 2021) that uses light in the form of pulsed lasers to measure distances to objects. It has become an integral technology in various industries, including autonomous vehicles (Elhousni and Huang, 2020), robotics (Mochurad et al., 2023a), and environmental monitoring (Guo et al., 2020).

Lidar systems consist of three main components:

1. *Laser transmitter:* This component generates short pulses of laser light (usually in the infrared) that are directed at objects in the environment.
2. *Detector:* The detector receives reflected light pulses from objects and converts them into an electrical signal.
3. *Data processing system:* The data processing system calculates the distance to objects using the time elapsed between the transmission of the pulse and the receipt of the reflected signal. Using the known angles and orientation of the Lidar system, the coordinates of the reflected points in three-dimensional space can be determined.

Lidar can be:

1. *Static Lidar:* Used to scan static objects from a fixed position. This is often used in surveying and mapping to create three-dimensional models of the landscape and infrastructure.
2. *Mobile Lidar:* Used to collect data when the Lidar system is on a moving object, such as a car, drone, or airplane. Mobile Lidar provides fast data collection over large areas and wide coverage. It is used in industries such as aerial surveying, infrastructure monitoring, and autonomous vehicles in particular.

However, Lidar has some disadvantages, such as high cost, relatively large size and weight, and sensitivity to weather conditions such as rain or fog. Some of these disadvantages can be compensated for by combining Lidar with other technologies and developing new, more compact and cost-effective Lidar systems.

One of the key applications of lidar is localization, which involves estimating the position and orientation of an object in the environment using data from a lidar sensor (Marck et al., 2013). Localization is extremely important for autonomous vehicles (Lu et al., 2022), where it is necessary to determine the position of the vehicle for safe and efficient operation. Optimization of navigation algorithms and methods can contribute to environmental and economic development, as autonomous vehicles can reduce fuel costs and ensure efficient use of infrastructure (Varsi et al., 2021).

Improving the navigation algorithms of autonomous cars can accelerate the development of smart cities, where autonomous vehicles play an important role in creating integrated and efficient transportation solutions (Phang et al., 2021; Wang et al., 2022).

Lidar’s localization speed is an important factor for real-time applications (Liu et al., 2023), especially for applications such as autonomous driving (Luo et al., 2019) where timely decision making is essential. Traditional localization methods such as the Extended Kalman Filter (EKF) (Zhang, 2019) and the iterative closest point algorithm (ICP) (Zhang et al., 2022), can be computationally expensive and do not meet the requirements of real-time applications (Dabbiru et al., 2020; Shymanskyi et al., 2022).

As it is known (Garland et al., 2008), the CUDA parallel computing platform was developed by NVIDIA, which can significantly accelerate various applications, including Lidar localization. CUDA allows developers to use the massively parallel architecture of modern GPUs, which allows them to process large amounts of Lidar data faster.

The relevance of the conducted research can be considered from the following perspectives:

• *Development of autonomous vehicles:* With the active growth of the autonomous vehicle industry, the development of new and improvement of existing navigation methods are becoming increasingly relevant. Autonomous vehicles require high accuracy in localization and stable operation of navigation algorithms for safe and efficient movement.
• *Improved traffic safety:* Enhancing the localization methods of autonomous vehicles will contribute to ensuring a high level of safety for passengers, pedestrians, and other road users, thereby reducing the risk of accidents and collisions on the roads.
• *Environmental sustainability and cost-effectiveness:* Optimizing navigation algorithms and methods can contribute to both environmental and economic development, as autonomous vehicles have the potential to reduce fuel costs and ensure efficient use of infrastructure.
• *Application in various fields:* Improvement of navigation algorithms can have a positive impact on various sectors, including logistics, automated warehouses, and robotics, where high-precision localization and navigation are critically important for efficient operations.
• *Advancement of artificial intelligence technologies:* The use of artificial intelligence methods, such as machine learning and computer vision, enables the creation of more accurate and adaptive navigation systems that can autonomously improve over time during operation.
• *Application of parallel computing:* The use of parallel computing significantly enhances the speed of algorithms and ensures more efficient processing of large volumes of data received from the sensors of autonomous vehicles.
• *Integration with other transportation systems:* Improving the navigation system of autonomous vehicles can facilitate integration with other transportation systems, such as intelligent road networks and public transportation systems.
• *Development of smart cities:* Enhancing the navigation algorithms of autonomous vehicles can expedite the development of smart cities, where autonomous transport plays a crucial role in creating integrated and efficient transportation solutions.
• *Ensuring transportation accessibility:* Improving the accuracy of autonomous vehicle localization can help ensure transportation accessibility for individuals with disabilities, the elderly, and other population groups for whom independent car operation may be difficult or impossible.
• Enhancing the competitiveness of automakers: The development and optimization of navigation algorithms can help automakers increase their competitiveness in the market by offering consumers autonomous vehicles with high precision in localization and navigation. This can contribute to the advancement of autonomous transport and the widespread adoption of these technologies among a broad range of users.

The results of this research can have a positive impact on road safety, cost-effectiveness, environmental sustainability, and transportation accessibility. Additionally, they can contribute to the development of smart cities, integration of transportation systems, and the enhancement of competitiveness for automakers.

The relevance of employing parallel computing in the context of autonomous car navigation becomes evident when considering the following factors:

1. *Large Data Volumes* (Huang and Cao, 2021): Autonomous vehicles accumulate substantial data from diverse sensors like lidars, radars, and cameras. Swift processing of this data is crucial for appropriate responses to varied situations. Parallel computing enables simultaneous data processing, enhancing the efficiency of the navigation system.
2. *Algorithmic Speed* (Varsi et al., 2020): The swift execution of navigation tasks such as localization, route planning, and obstacle detection is imperative for autonomous cars. Parallel computing facilitates the distribution of tasks across numerous processors or cores, resulting in rapid responses and reduced information processing durations.
3. *Energy Efficiency* (Bi et al., 2020): Through optimal allocation of computing resources, parallel computing contributes to more energy-efficient navigation algorithms. This aspect is particularly significant for electric and hybrid vehicles with limited energy resources.
4. *Compatibility with Distributed Systems* (Amin et al., 2019)*:* Parallel computing can be leveraged to create distributed data processing systems. Different segments of navigation algorithms can be executed on diverse devices or nodes within the computing network, optimizing overall system performance, reliability, and scalability.
5. *Real-Time Assurance* (Mochurad and Shchur, 2021): Given the necessity for autonomous vehicles to respond to traffic situations in real-time, parallel computing plays a crucial role in ensuring swift execution of algorithms. This is essential for maintaining safe and efficient traffic conditions.
6. *Adaptability to Various Computing Resources* (Huang and Cao, 2021)*:* Parallel computing is applicable across different computing platforms, including CPUs, GPUs, and specialized accelerators like FPGAs and ASICs. This adaptability allows navigation algorithms to be tailored to the available resources, optimizing their overall efficiency.

The article analyzes the literature on the topic of the study. This was done with a view to highlighting the main advantages and disadvantages of the current state of the issue under consideration. In the paper (Montañez et al., 2023), the authors employed an extended Kalman filter for the detection of moving objects. The effectiveness of the EKF was assessed using a dataset that includes location information obtained from LiDAR and a radar sensor for an object moving along a trajectory with abrupt changes.

In (Koide et al., 2021) presents an approach that creates a globally consistent 3D map structure based on the loss factor during a real-time GPU-accelerated mapping process. Data is obtained from a 3D Lidar and maps are constructed based on it. The GPU is used to speed up the mapping algorithm during map creation.

In the research (Shreyas Madhav and Rajesh Kanna, 2021), an advanced Lidar 3D SLAM algorithm is introduced for autonomous aerial robots. The alignment process involves the extraction of Fast Point Feature Histogram (FPFH) descriptors, subsequently refined through iterative nearest point registration (NPR). The ultimate trajectory estimation undergoes 3D pose graph optimization to reduce potential overall drift. Simulated results demonstrate a noteworthy 26% decrease in execution time when employing the parallelized algorithm with 4 CPUs compared to its serial counterpart.

In (Jang et al., 2022), the authors detail an algorithm that employs GPU parallel processing to enhance the existing ND map matching process. This optimization resulted in a remarkable 48-fold acceleration while preserving accuracy.

The integration of a semantic image with low-resolution 3D Lidar point clouds and the generation of dense semantic depth maps are addressed in (Lou et al., 2023). Utilizing visual odometry, the method selects functional ORB points with depth information to enhance positional accuracy. During unmanned vehicle positioning, parallel threads are employed to aggregate 3D semantic point clouds.

In the paper (Chiang et al., 2023), the authors leverage Lidar as the primary auxiliary sensor, proposing a Lidar-based simultaneous localization and mapping (SLAM) approach for positioning, navigation, and synchronization. Furthermore, point cloud registration is executed through a three-dimensional normal distribution transform (NDT). The initial Lidar position assumption for Lidar-based SLAM is derived from two sources: one being a differential global navigation satellite system (GNSS) solution, and the other being an inertial navigation system (INS) and an integrated GNSS solution created using an extended Kalman filter with added motion constraints, including zero velocity update and nonholonomic constraint.

An improved NDT algorithm and its FPGA implementation were presented in (Deng et al., 2021). The authors achieved the acceleration of the search operation by using a new data structure called OAVS, which is non-recursive and efficient. The optimized semantic NDT algorithm based on OAVS significantly reduced the number of search operations by eliminating unnecessary queries. Additionally, the proposed streaming FPGA accelerator architecture for SEO-NDT improved real-time performance and ensured energy efficiency. When compared to advanced embedded CPU and GPU processors, the FPGA implementation provided up to 35.85x and 2.44x performance acceleration, respectively.

In (Dong et al., 2021) authors used this method in such a way that it performs all calculations directly on the range images created using 3D LiDAR scans, which avoids explicit processing of the 3D point cloud and quickly selects the poles for each scan.

As indicated in (Mendez Maldonado et al., 2021), the authors developed a hybrid convolutional neural network (CNN) by directly applying a Markovian grid-based localization approach on the GPU. This CNN is capable of simultaneously handling image-based localization and odometry-based probability propagation within a single neural network. The detailed description of the Markovian approach can be found in (Kovtun et al., 2023a).

In (Sun et al., 2020) a new data structure with a spatial partitioning method was presented, which can be successfully built even for large volumes of point clouds. Based on this structure, a KNN search algorithm was developed that works effectively when the distribution of points is uneven. This innovative structure is implemented on both an FPGA accelerator and a GPU.

In the following paper (Xie et al., 2022), introduces a lightweight convolutional neural network (CNN) framework designed for the semantic segmentation of a projection-based LiDAR point cloud. This framework comprises only 1.9 million parameters, marking an 87% reduction compared to leading-edge networks. The evaluation on a GPU revealed a processing time of 38.5 milliseconds per frame and an achieved result of 47.9% mIoU on the Semantic-KITTI dataset. Moreover, the proposed CNN is tailored for FPGAs using the NVDLA architecture, demonstrating a 2.74x speedup compared to a GPU-based implementation and a noteworthy 46x improvement in energy efficiency.

In (Mochurad and Kryvinska, 2021) a parallel parallelization algorithm is proposed to solve the problem of determining the current position of a lidar in 2D based on OpenMP technology. The authors also indicated prospects for further research: 1) optimization of the computing process based on CUDA technology using GPUs; 2) consideration of a more complex spatial domain.

The researchers in (Mochurad et al., 2023b) introduced a parallel algorithm employing CUDA technologies to establish the 2D position of a lidar through the Particle Filter algorithm. Despite achieving a considerable speedup with this technology, it might appear that their findings challenge the hypothesis presented in our study. Nonetheless, this is not the case, as our investigation focuses on a distinct algorithm, addressing the issue of 3D localization and extending beyond closed-room localization.

In the study (Xu et al., 2022), the use of measurement uncertainty estimation is identified as an effective method for tracking a vehicle, based on LiDAR detectors. The authors propose an extended Kalman filter framework, consisting of two main components: the first is capable of assessing the statistics of measurement noises dependent on the state to detect LiDAR objects, while the second generates multi-hypothesis measurements based on the trajectory of the identified vehicle.

The shift from traditional automobiles to autonomous ones encompasses the integration and enhancement of diverse technologies and computerized algorithms. An integral aspect influencing the efficacy of autonomous vehicles is their localization, along with perception, route planning, and control, where the precision and effectiveness of localization assume a pivotal role in autonomous driving. About (Poulose et al., 2022), the paper underscores the significance of the localization challenge in autonomous vehicles and elucidates its map-based realization employing point cloud matching. The authors introduce a localization system leveraging the Robot Operating System (ROS) in conjunction with Autoware. The empirical findings demonstrate that a map-centric localization system utilizing 3D lidar scanning delivers adequately precise real-time localization for autonomous driving within a university campus setting. The paper provides an exhaustive account of the methodologies for crafting point cloud maps and vehicle localization, along with a systematic guide for implementing a map-based system for autonomous driving.

In cities, there are many regions where the global navigation satellite system does not work, where localization of autonomous driving remains a problem. Various methods have been previously proposed to improve the localization accuracy by using accurate distance measurements obtained from Lidar sensors and for the speed of map construction. This study proposes a parallelized 3D Kalman algorithm using CUDA to accelerate the computational speed of Lidar localization while maintaining the original lidar localization accuracy. Unlike previous papers that parallelize the map construction, this approach parallelizes the Kalman localization algorithm itself.

The aim of this study is to propose a parallel algorithm based on CUDA technology to accelerate lidar localization in 3D space.

The main contribution of this article can be summarized as follows:

1. Anew localization algorithm is proposed that uses the Kalman filter and CUDA technology to accelerate the computational speed of Lidar localization in 3D;
2. A theoretical estimate of the acceleration based on Ambdahl’s law was calculated;
3. A comparison of the sequential Kalman algorithm and the parallel implementation for different sizes of datasets is carried out, and quantitative estimates of the advantages obtained over existing studies are given;
4. The localization error is determined, and it is found that the proposed algorithm allowed to obtain a speedup of 3.8 times without reducing the localization error, which amounted to 3%.

The rest of this article is organized as follows: Chapter 2 describes the proposed parallel localization algorithm, analyzes the computational complexity presents a new theoretical estimate of the speedup based on Ambdahl’s law, and presents the steps of implementing the proposed algorithm using CUDA technology. Chapter 3 describes the environment used for testing and presents the relevant results of numerical experiments. Conclusions and prospects for further research are shown in the last chapter.

## 2 Materials and methods

The Kalman filter, discussed in (Wo and Biswal, 2023), serves as a powerful recursive tool for estimating the internal state of linear dynamic systems by analyzing a series of noisy measurements. Its application extends across various domains, encompassing engineering, economics, radar, computer vision, and the estimation of structural macroeconomic models. This filter holds significance as a fundamental component in control theory and the development of control systems. In conjunction with the linear-quadratic regulator (LQR), the Kalman filter addresses the challenges posed by linear-quadratic Gaussian (LQG) control problems. Collectively, the Kalman filter, LQR, and LQG controller represent essential solutions to core issues in control theory.

As noted by the authors of (Meng et al., 2023), Kalman filtering is an optimal recursive numerical computing algorithm characterized by the efficiency of program memory use, speed, and suitability for real-time data processing programs.

It is based on a mathematical model of the system and uses the principles of Kalman filtering to combine the predicted state with actual measurements to obtain the best estimate of the system’s state. It has two main steps: prediction and correction. The prediction step uses mathematical models of the system and the previous state estimation to make a forecast of the future state of the system. In the correction step, the predicted state is updated to take into account new measurements that are reduced by noise and incompleteness. The state estimation uses information such as the state vector and covariance matrix to provide an optimal estimate and minimal prediction error. This is achieved by weighting the prediction and new measurements based on their accuracy and uncertainty.

Kalman filters are constructed upon time-discretized linear dynamical systems, which are represented as Markov chains, as outlined in (Kovtun et al., 2023b). These chains are built on linear operators subject to errors, which may include Gaussian noise. The system’s state is expressed as a vector of real numbers. At each discrete time increment (clock cycle), a linear operator is applied to the state, generating a new state that incorporates some noise and, if available, information from the system control. Subsequently, another linear operator, combined with additional noise, is applied to the true (“hidden”) state to produce the observed outputs. While the Kalman filter shares similarities with the hidden Markov model, a key distinction lies in the fact that the hidden state variables in the Kalman filter assume values in a continuous space, contrasting with the discrete state space of the hidden Markov model. Notably, there exists a robust duality between the equations of the Kalman filter and the hidden Markov model.

• Foresight stage

**FIGURE 1:**
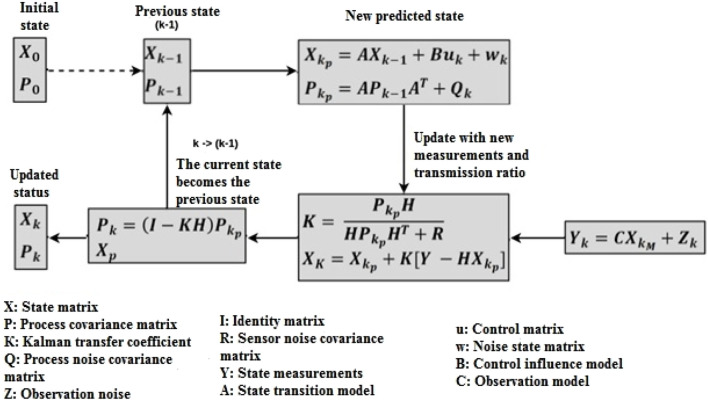
Flowchart of the multivariate Kalman filter algorithm.

The Kalman filter model postulates that the actual state at a given time point k is deduced from the state at k−1 , as illustrated Figure 1:

$$X_{k_{p}}=AX_{k-1}+Bu_{k}+w_{k}$$

where.

$A_{k}$ is a state transition model applied to the previous state $x_{k-1}$ ;

$B_{k}$ is a model of control effects applied to the control vector $u_{k}$ ;

$w_{k}$ is the noise of the process, which is assumed to have a multivariate normal distribution with zero mean and covariance $Q_{k}$ .

The covariance of the predicted $P_{k}$ state is calculated using the following formula:

$$P_{k_{p}}=AP_{k-1}A^{T}+Q_{k}\;.$$

• Refinement stage

At a point in time k observation (or measurement) $Y_{k}$ of the present state $X_{k}$ is made in accordance with

$$Y_{k}=CX_{k_{m}}+Z_{k}$$

where C is the observation model that maps the true state space to the observed space, and $Z_{k}$ is the observation noise, assumed to be Gaussian white noise with zero mean and covariance $R_{k}$ .

Next, the covariance of innovations (deviation) is calculated $S_{k}$ which is then used to calculate the optimal Kalman transfer coefficient:

$$S_{k}=H_{k}P_{k|k-1}H_{k}^{T}+R_{k}$$

$$K_{k}=P_{k|k-1}H_{k}^{T}S_{k}^{-1}$$

After that, we calculate the updated state estimate and its covariance:

$$X_{k}=X_{k_{p}}+K\left[Y\;-\;HX_{k_{p}}\;\right]$$

$$P_{k}=\left(I\;-\;KH\right)P_{k_{p}}$$

The initial state and noise vectors at each cycle { $x_{0}$ , $w_{1}$ , $w_{k}$ , $v_{1}$ . $v_{k}$ } are assumed to be mutually independent.

### 2.1 The proposed parallel algorithm description

CUDA was used to parallelize the Kalman algorithm. Since all the operations in the Kalman algorithm are vector operations, i.e., transformations and other calculations are performed by matrix operations, it was decided to speed up their execution by moving them to CUDA. Thus, we have two subtasks: the prediction stage and the refinement stage. We created corresponding execution kernels for them. Next, we present an overview of the CUDA-based Kalman algorithm system:

1. *Input Data*:
  • The algorithm takes input data related to the prediction and refinement stages. This may include state predictions, covariance estimates, and other relevant information for each point.
2. *Prediction Stage Kernel*:
  • CUDA kernel specifically designed for the prediction stage.
  • Parameters: gridSize = (point_num, 1), blockSize = (Predict_Size, Predict_Size).
  • Each thread processes a point, calculates state prediction $X_{k_{p}}$ , and covariance $P_{k}$ using the transition matrix A .
  • Execution involves parallel matrix operations for multiple points.
3. *Synchronization (Prediction Stage)*:
  • Threads are synchronized after completing calculations for the current step.
  • Ensures all threads have updated values of assumptions and covariances for the next steps.
4. *Refinement Stage Kernel*:
  • CUDA kernel dedicated to the refinement stage.
  •Parameters: gridSize = (point_num, 1), blockSize = (Predict_Size, Predict_Size).
  • Each thread processes a point, calculates deviation covariance $S_{k}$ , optimal Kalman transfer coefficient $K_{k}$ , updates assumptions $X_{k}$ , and covariance $P_{k}$ .
  •Execution involves parallel matrix operations for multiple points.
5. *Matrix Inversion (Refinement Stage)*:
  • As part of calculating $K_{k}$ , matrix inversion is required.
  • All threads are synchronized to perform matrix inversion collectively.
  • After inversion, parallel calculations resume.
6. *Synchronization (Refinement Stage)*:
  • Threads are synchronized again after calculating each update element.
  • Ensures consistent updated values before moving to the next step of the algorithm iteration.
7. *Output Data*:
  • The algorithm produces updated assumptions and covariances after both prediction and refinement stages.

This presents an overview of the CUDA-based Kalman algorithm system and provides an overview of how the CUDA-based Kalman algorithm processes input data, performs parallelized matrix operations, and synchronizes threads at critical points to maintain consistency in the calculations.

### 2.2 Analysis of computational complexity and theoretical estimation of speedup

The complexity of the Kalman algorithm:

$$O\left(n_{z}^{2.4}+n_{x}^{2}\right),$$

where $n_{x}^{2}$ follows from the manipulation of the matrices by the dimension $n_{x}$ x ${\;n}_{x}$ , а $n_{z}^{2.4}$ to the power of 2.4 due to the inversion of the matrix $n_{z}$ x $n_{z}$ .

Therefore, in the context of parallelization using CUDA, the time complexity will be equivalent to the algorithm’s complexity divided by the number of threads, with the exception of matrix inversion, as it involves synchronization.

$$O\left(n_{z}^{2.4}+\frac{n_{x}^{2}}{N}\;\right),$$

where N is the number of threads.

Since the Kalman algorithm consists of matrix operations, it was fully parallelized, but the matrix inversion is performed in synchronous mode. The data reading operations can be neglected, so for the first Amdahl’s law (Anshu, 2019), the value of the sequential part (α) for the Kalman filter position and velocity prediction problem can be assigned the ratio of the complexity of the parallelized matrix operations to all other operations, namely,:

$$\alpha =\frac{n_{z}^{2.4}}{n_{z}^{2.4}+{6n}_{x}^{2}}\approx \frac{73,72}{289.72}\approx 0.26$$

Then the theoretical acceleration is as follows:

$$S_{p}=\frac{1}{\alpha +\frac{1-\alpha }{p}}=\frac{1}{0,26+\frac{1-0,26}{640}}\approx 3.9,$$

where *α* is the fraction of the sequential algorithm, *p* is the number of cores.

### 2.3 Implementation of the proposed algorithm

CUDA was used to parallelize the Kalman algorithm. Since the Kalman algorithm consists of matrix operations, it was fully parallelized. That is, all matrix operations from the prediction and update stages were encapsulated in two corresponding processing kernels on the GPU memory. This way, we got a kernel for predictions and updates, which made it possible to speed up the matrix operations of the algorithm itself, i.e., the algorithm itself.

Since the algorithm is iterative, after all the cores have completed the operation of one pass, synchronization was established to avoid possible situations of resource races or access to uncalculated values.

To use CUDA, we built two processing functions for each of the stages. The function of the prediction stage is performed as follows:

1. Thread indexing: The **tx** and **ty** variables store the index of the thread in the block, and **bx** stores the block index.
2. Allocation of shared memory: By using the **__shared__** keyword, a shared memory location is declared for the **temp** array with the size of the covariance matrix used for intermediate results.
3. State prediction: each thread calculates the predicted state ${x_{k}}^{\prime }$ for a particular point using the formula $x_{k}^{\prime }=A∙x_{k-1}$ where the required range of matrices is selected for each specific point. This calculation is performed only by the first thread in each block (**if (tx < 1)**), and the results are stored in the **new_predictD** array.
4. Prediction of covariance: Each thread computes the predicted covariance matrix ${P_{k}}^{\prime }$ for a particular matrix element using the following formula $P_{k}^{\prime }=A∙P_{k-1}∙A^{T}+Q$ . The intermediate result is stored in the shared array **temp.**
5. Update covariance: The intermediate results stored in **temp** are multiplied by $A^{T}$ each individual element in its own stream. To each element of the final result is added the corresponding element of the matrices Q i. These calculations are performed in the condition (**if (bx < point_num**) to avoid a possible access attempt outside the allocated memory. The result is saved to the **new_covD** array.
6. Synchronization of threads: The **__syncthreads()** function ensures that all threads in a block complete their calculations and synchronize them before continuing execution.

The refinement function is performed according to the algorithm described below:

1. Indexing of threads: The **tx** and **ty** variables store the indexes of threads within the same block, **bx** stores the block number.
2. Allocation of shared memory: The **__shared__** code word declares shared memory for the arrays **temp**, **temp2**, **temp3**, K , **temp4,** and **temp5** for intermediate calculations and the value of the Kalman coefficient.
3. Calculations. $H∙P_{k}$ : Each thread computes one specific element of the product of two matrices $H∙P_{k}$ using the required set of values. The result of each thread is stored in a shared array **temp.**
4. Calculations. $H∙P_{k}H^{T}+R$ : Streams compute a specific single element of the result of an operation $H∙P_{k}H^{T}+R$ formula using the value already found $H∙P_{k}$ on the previous one. The result is stored in the shared array **temp2.**
5. Calculations. $P_{k}H^{T}$ : Each thread computes one specific element of the result of the product $P_{k}H^{T}$ . The result is stored in the shared array **temp3.**
6. Synchronization and search for the inverse matrix: The threads are synchronized so that all previous operations have finished their calculations and the subsequent execution is with a fully filled **temp2** array. To find ${\left(H∙P_{k}H^{T}+R\right)}^{-1}$ the already calculated value of the matrix is taken and the value of the inverse is searched for on its basis in sequential mode. The result of the inverse matrix search is stored in **temp2_inv.**
7. Calculation K: Threads calculate one specific element of the Kalman transfer coefficient matrix using the calculation results stored in **temp3** and **temp2_inv.** The result is saved to a shared array K .
8. Calculations. $z_{k}-H∙{x_{k}}^{\prime }$ : Each thread computes one specific difference element $z_{k}-H∙{x_{k}}^{\prime }$ . The result is stored in **temp4.**
9. Calculations. $x_{k}$ : Threads compute one value of the refined prediction of the next state at a time $x_{k}$ by adding the product of K by $z_{k}-H∙{x_{k}}^{\prime }$ stored in temp4, to the initial state prediction.
10. Calculations. I−K∙H : Threads compute one specific element of the matrix I−K∙H . The result is stored in **temp5.**
11. Calculations. $P_{k}$ : The threads compute the updated covariance matrix $P_{k}$ by multiplying I−K∙H stored in **temp5**, with the initial value of the covariance matrix P .
12. Synchronization of threads: The **__syncthreads()** function ensures that all threads in a block complete their calculations and synchronize them before continuing execution.

So, Algorithm 1 described implementing the proposed algorithm.

 //Initialization of constant variables and matrices
 //const Predict, Measure, PredictSize, CovSize, MeasureSize
 //H, HT, A, AT, Q, R, I
 function ele_multi (A, B, Awidth, Bwidth, tx, ty):
  P, k = 0, 0
  for k = 0 to Awidth: P + = A [ty * Awidth + k] *B [k * Bwidth + tx]
  return P
 function inv_cpu (a_i, c_o, n):
  d = 0, n = 3
  for i = 0 to 3: d + = a_i [0 * 3 + i] * (a_i [1 *n + ((i + 1) % 3)] * a_i [2 *n + ((i + 2) % 3)] - a_i [1 *n + ((i + 2) % 3)] * a_i [2 *n + ((i + 1) % 3)])
  if d: for i = 0 to 3: for j = 0 to 3: c_o [i *n + j] = ((a_i [((j + 1) % 3) *n + ((i + 1) % 3)] * a_i [((j + 2) % 3) *n + ((i + 2) % 3)]) - (a_i [((j + 1) % 3) *n + ((i + 2) % 3)] * a_i [((j + 2) % 3) *n + ((i + 1) % 3)]))/d
 function PredictKernel (predictD, covD, new_predictD, new_covD, point_num):
  for bx = 0 to point_num:
  for tx = 0 to Predict: new_predictD [bx * PredictSize + tx] = ele_multi (A, predictD + bx * PredictSize, Predict, 1, tx, 0), temp [0][tx] = ele_multi (A, covD + bx * CovSize, Predict, Predict, tx, 0)
  __syncthreads ()
  new_covD [bx * CovSize + tx] = ele_multi (temp, AT, Predict, Predict, tx, 0) + Q [tx],
  __syncthreads ()
  function UpdateKernel (dataD, predictD, covD, new_predictD, new_covD, point_num, ite_num):
  for bx = 0 to point_num:
   for ty = 0 to Measure: temp [ty][0] = ele_multi (H, covD + bx * CovSize, Predict, Predict, 0, ty)
   __syncthreads ()
   for ty = 0 to Measure: for tx = 0 to Measure: temp2 [ty][tx] = ele_multi (temp, HT, Predict, Measure, tx, ty)
   __syncthreads ()
   for tx = 0 to Measure: temp3 [0][tx] = ele_multi (covD + bx * CovSize, HT, Predict, Measure, tx, 0)
   __syncthreads ()
   for ty = 0 to 2: for tx = 0 to 1: temp2_inv [ty * Measure + tx] = ele_multi (temp2_inv_f, temp3, Measure, Measure, tx, ty)
   __syncthreads ()
   for ty = 0 to Measure: temp4 [ty] = dataD [MeasureSize * bx + ty] - ele_multi (H, predictD + bx * PredictSize, Predict, 1, 0, ty)

Algorithm 1 Parallelized the Kalman algorithm.

   if tx = = 0: new_predictD [bx * PredictSize +0] = predictD [bx * PredictSize +0] + ele_multi (K, temp4, Measure, 1, 0, 0)
   temp5 [0][0] = I [0][0] - ele_multi (K, H, Measure, Predict, 0, 0), __syncthreads ()
   new_covD [bx * PredictSize + tx] = ele_multi (temp5, covD + bx * CovSize, Predict, Predict, tx, 0)

## 3 Results

For the study, a random three-dimensional space of values was generated (Top Streamers on Twitch, 2023) since the object of study is the speed of the algorithm. Random values were generated taking into account the contribution of measurement error and had a nonlinear characteristic.

The tests were conducted on a system with the following characteristics:

### 3.1 System

CPU: core i5-8500H.

RAM: DDR4 2,667 MHz 24 Gb.

GPU: NVIDIA GeForce GTX 1050.

GPU RAM: GDDR5 8192 MB.

CUDA Cores: 640.

### 3.2 Memory interface: 256-bit

GPU Interface: PCI Express x8 Gen3.

As a result of applying the approach proposed in this paper, the execution times of the parallelized algorithm using CUDA and the algorithm implemented on the CPU are presented in Table 1.

**TABLE 1:** Execution time, s.

Number of points	GPU	CPU
10	1.074	0.334
50	1.589	1.568
100	2.695	2.957
500	6.350	15.227
1,000	8.295	29.450
2000	16.253	58.511
5,000	38.609	146.716
10,000	77.652	295.801

**FIGURE 2:**
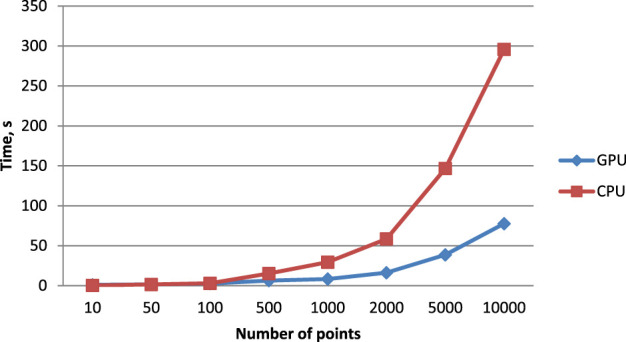
Visualization of the execution time of the proposed algorithm on GPU using CUDA and on the CPU.

For visualization, the data in Table 1 are presented in Figure 2. According to the results shown in the table, for a small number of points, up to 50, the single-threaded CPU algorithm performs the same number of operations faster, because there are not enough points to optimally load the GPU and the time spent by CUDA on memory allocation is longer than the time of computation.

Based on the data in Table 1, the value of the resulting acceleration S which is shown in Table 2.

**TABLE 2:** Acceleration of the parallel Kalman algorithm when using CUDA relative to CPU.

Number of points	10	50	100	500	1,000	2000	5,000	10,000
S	0.310	0.986	1.097	2.397	3.550	3.600	3.800	3.809

**FIGURE 3:**
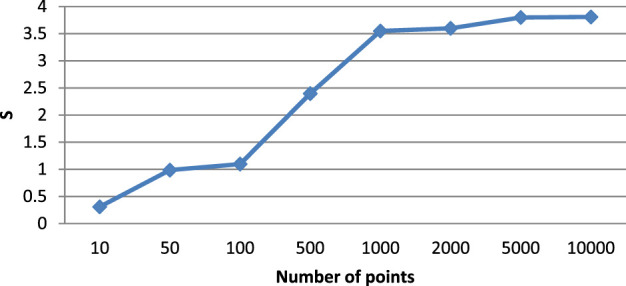
Acceleration of the parallel Kalman filter algorithm.

The results in Table 2 show that the speedup reaches its threshold under the given conditions by 3.8. The reliability of this result, obtained based on numerical experiments of the software implementation of the proposed algorithm, is confirmed by the previously obtained theoretical estimate of the speedup, which should be equal to 3.9. Thus, there is a speedup after 500 points, but before that the algorithm is slower than the traditional one. These results are also visualized in Figure 3. As you can see, the algorithm gets close to the maximum speedup from 1,000 to 5,000 points. And then it slowly rises.

Thus, the software implementation of the proposed algorithm made it possible to process object location data up to 3.8 times faster. This acceleration, in turn, makes it possible to build real-time systems that require fast localization processing. Such systems can be autonomous vehicles or car pilot assistance systems, where the fastest possible processing of frequently received data on position in space and environment is required. The high speed of data flow is optimally suited for processing on CUDA.

**FIGURE 4:**
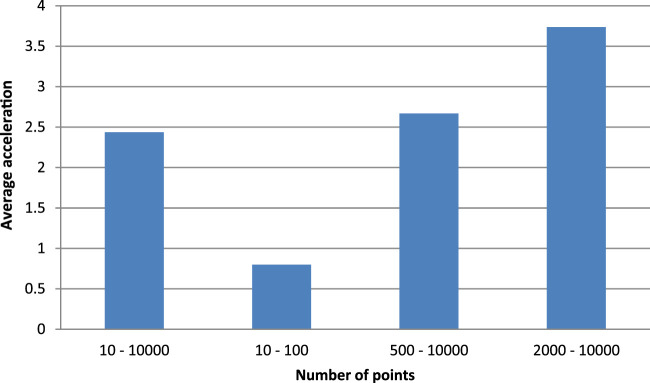
Comparison of average GPU acceleration for different datasets.

In order to compare the sequential Kalman algorithm and the parallel implementation, tests and measurements were performed for different sizes of item datasets. These data were averaged and presented in a report for further analysis. Based on the results of the tests of the sequential and parallel Kalman algorithms, a graph comparing the average acceleration over different ranges of the number of points was constructed, which is shown in Figure 4.

According to Figure 4, we can conclude that it is more efficient to delegate localization using the Kalman algorithm to a larger GPU, from 500 points. As we can see from the averaged results, the use of CUDA on average gives an increase in execution speed of about 2.4 times. While when using the optimal set, it is 3.7 times faster. It can also be seen that using only suboptimal sets leads to losses in execution speed, as due to the previously described features of the algorithm and technology, the acceleration is only 80% of the typical CPU sequential algorithm.

As a result, we obtained a variation of the Kalman algorithm for localization in 3D space. The proposed approach is appropriate only for systems with a high load of data flow for localization, which will speed up localization by 3.8 times. It may be inappropriate to use it instead of the traditional approach when there are long delays between new data acquisitions, as the execution speed will not increase. Accuracy for both variations of the localization algorithm is shown in Table 3. As can be seen from the results, the quality of localization is almost identical for both algorithms. According to the results, both implementations have an average localization error of 3%, which indicates a shift from the real position by no more than 3% from the original position.

**TABLE 3:** Localization error.

Number of points	Localization error, %	
	GPU	CPU
10	3.156481147	3.121895142
50	3.26348609	3.341513087
100	2.829944308	2.818013953
500	2.726926206	2.721352679
1,000	3.087015553	3.086803368
2000	2.995234731	3.058086489
5,000	3.067066336	3.161766167

Thus, the proposed algorithm allows us to obtain a solution 3.8 times faster without reducing the localization error, which is effective in real-time decision making.

The results obtained were compared with the previous results obtained by other authors. For example, in (Jonsson, 2012), the author obtained a speedup of 1.5 for the number of points of 500–10000, while in our work this result was improved by 60%. Also, compared to (Osman et al., 2021), we managed to improve the speedup by about 90%. In (Sheikhpour and Atia, 2022), the authors managed to speed up the processing time of a parallel algorithm by 41% compared to a conventional sequential implementation, while we managed to do it by 73%.

## 4 Conclusion

In this paper, we developed a parallelized version of the Kalman algorithm in 3D using CUDA to accelerate the computational speed of Lidar localization. Localization using Lidar is relevant for autonomous driving in regions where the global navigation satellite system does not work. The result is a software product that processes object location data faster and can be used for real-time systems, such as autonomous vehicles or car pilot assistance systems, where frequently received data on position and environment need to be processed as quickly as possible. High data rates are optimally suited for CUDA processing.

We tested and measured the efficiency of the sequential Kalman algorithm and the parallel implementation on different sizes of lidar position datasets. It turned out that the use of CUDA is more efficient on larger datasets, from 500 points. On average, using CUDA gives an increase in execution speed of about 2.4 times, while on the optimal set – 3.8 times. Using CUDA on suboptimal sets can lead to losses in execution speed, since due to the peculiarities of the algorithm and acceleration technology, the execution speed is only 80% of the sequential version. This can be explained by the fact that CUDA allows you to calculate many points in parallel at the same time, which reduces the execution time of the algorithm.

In general, the use of CUDA can significantly increase the efficiency of the Kalman algorithm, because in real conditions, it is necessary to constantly process the data coming from the lidar. Nowadays, there are many CUDA-enabled edge devices, which confirms the relevance of the presented algorithm and this study.

Prospects for further research include the possibility of extending the proposed algorithm to the case of 2D LIDAR data and analyzing the proposed algorithm based on SLAM technology (Zhu et al., 2009).

## Data Availability

The datasets presented in this study can be found in online repositories. The names of the repository/repositories and accession number(s) can be found in the article/Supplementary material.
